# Kernel-based formulation of intervening opportunities for spatial interaction modelling

**DOI:** 10.1038/s41598-020-80246-9

**Published:** 2021-01-13

**Authors:** Masaki Kotsubo, Tomoki Nakaya

**Affiliations:** Graduate School of Environmental Studies, Tohoku University, 468-1, Aoba, Aramaki, Aoba-ku, Sendai-city, Miyagi, 980-0845 Japan

**Keywords:** Mathematics and computing, Psychology and behaviour, Applied physics

## Abstract

Understanding spatial interactions such as human mobility has been one of the main analytical themes in geography, spatial economics, and traffic engineering for a long time. The intervening opportunities models, including the radiation model, provide a framework to elucidate spatial interactions generated by an individual’s distance-ordered decision-making process. However, such classical definitions of intervening opportunities have often failed to predict realistic flow volumes, particularly for short-distance flows. To overcome this problem, we have proposed a new formulation of intervening opportunities with a kernel function to introduce a fuzziness in spatial search behaviours of destinations, to develop a new variant of the radiation model. The mobility patterns resulting from the modified radiation model that included kernel-based intervening opportunities outperformed the original radiation model when fitted to four datasets of inter-regional flows.

## Introduction

The flow of people, freight, or information from one area to another is called spatial interaction, and represents the social links, connections, or relationships between them^1^. For many years, researchers have attempted to explain and model these interaction regularities and patterns^2–4^. Spatial interaction models, used to model the quantities of interregional flow, are generally formulated as $T_{ij} = f\left( {V_{i} , W_{j} , S_{ij} } \right)$, where $T_{ij}$ represents the size of flows from origin $i$ to destination $j$; $V_{i}$ and $W_{j}$ represent origin propulsiveness and destination attractiveness, respectively; $S_{ij}$ measures the spatial separation between the origin and destination areas. Although various specifications are possible for the spatial separation term, two types of formulations have been widely used: distance decay used in gravity models^5,6^, and intervening opportunities^7–9^.

Whereas the distance decay is derived from empirical laws^2,5,6^, the concept of intervening opportunities is rooted in spatial search behaviours of destinations for a moving individual. This is described by Schneider’s model, which assumes that when an individual searches for a destination, the individual will select an area that is closest to the origin among possible candidates having higher opportunity benefits than those of the origin^7,10^. For capturing the effects of spatial structure, i.e., of origin–destination locational patterns on aggregate inter-regional flow patterns^11–16^, several previous studies have attempted to incorporate intervening opportunities into gravity models to explicitly include their effects^17–20^.

The gravity and intervening opportunities models have sometimes been compared and these studies are not unanimous in their decisions on the best model for a variety of flows; sometimes the latter has performed equally with the former^21,22^, or outperformed the former^16,23–25^ and vice versa^26,27^. However, the intervening opportunities models are not frequently used because of the difficulties in handling the metrics with destinations ranked by the distance from the origin, and in the statistical estimation of parameters due to nonlinear formulation in the case of Schneider's model^10,28,29^.

The introduction of the radiation model^30^ triggered a new focus on the intervening opportunities models. The model has no free parameters to be estimated and has rigorous derivation based on the behavioural assumption, which is fundamentally the same as Schneider’s assumption of spatial search behaviour for a destination. Despite the high predictability for a wide range of flows including commuting, migration, and commodity in the United States, most of the later studies reported poor agreement between predicted and observed flows in different countries, which indicated that the radiation model could not universally predict human mobilities^31–39^.

To improve the performance of the radiation model, various efforts have been made through introducing additional parameters, reformulating the model to a more practical form^40–42^, and developing alternative models^38,43–47^. Although the remarkable feature of the radiation model is its simplification of the decision-making process as an analogy to particle emission and absorption to derive a parameter-free formulation, some researchers added parameters to reflect additional aspects in the decision-making process, such as a discretised settlement structure, spatial scale, and heterogeneity^40–42,45^. Regarding the geographical extent of defining intervening opportunities as the spatial separation $S_{ij}$, alternative distance metrics such as network distance, rather than Euclidean distance, were also proposed to ensure a realistic calculation of intervening opportunities^48^.

In previous studies, the radiation model generally tended to precisely predict volumes of flows for long distances, similar to inter-city flows^32,36,39,43,46^. Conversely, the results suggest that the radiation model fails to capture realistic travel patterns with short distance flows. Among these flows, there are many pairs of locations with approximately the same distance between them even after considering the direction of movements, and short distance flows have the main mass of the total size of flows, for example, in commuting^32^. Thus, an even subtle misspecification of which areas are included or excluded in the measurement of intervening opportunities can worsen the predictive accuracy of the radiation model.

In this study, we aim to introduce a new but simple operational scheme of intervening opportunities to improve the radiation model, using geographic kernel weights that allow a fuzzy extension of intervening opportunities, including when even farther places are considered compared to the destination. The scheme is derived from a decision-making process related to intervening opportunities and makes the distribution of intervening opportunities smoother, which may improve the radiation model’s performance, especially for short distance flows. We then tested the modified radiation model following the newly proposed formulation against four census mobility datasets and compared the performance of the new model with those of other models.

## Results

### The radiation model and a new formulation of intervening opportunities

**Figure 1:**
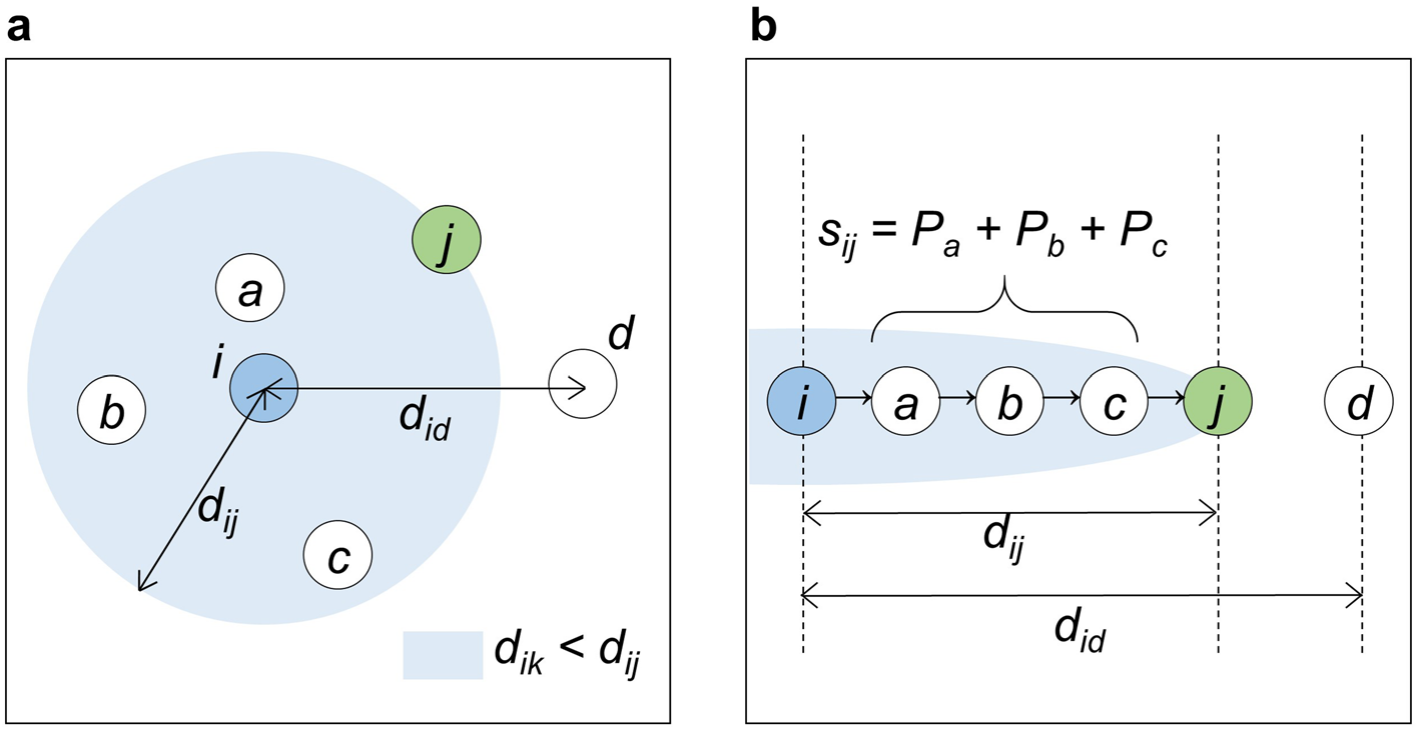
Schematic of intervening opportunities. (**a**) The geographical extent of intervening opportunities based on the distance between origin *i* and destination *j*. (**b**) The intervening opportunities $s_{ij}$ composed of the cumulative population of areas in nearby order in the geographical extent.

In the derivation of the radiation model^30^, when an individual seeks job offers from all locations, they first evaluate the benefit $z$ of the employment opportunities offered by these locations. Here, the number of opportunities in each area is assumed to be proportional to the area’s population, and the benefits of the opportunities are randomly chosen from a distribution $p\left( z \right)$. A spatial separation variable denoted as $s_{ij}$ is introduced as the total population of areas within the circle whose radius is the distance $d_{ij}$ between the origin $i$ and the destination $j$, centred at $i$ excluding the origin and destination populations as shown in Fig. 1. This is formulated as:

1 $$s_{ij} = \mathop \sum \limits_{{k \ne i,{ }j}}^{n} P_{k} I\left( {d_{ij} > d_{ik} } \right)$$

where $n$ is the number of destinations, $k$ is the area index of $k$th nearest neighbour to the area $i$, $P_{k}$ is the population of the area $k$, and $I\left( {d_{ij} > d_{ik} } \right)$ is the indicator variable which takes the value of 1 when $d_{ij} > d_{ik}$, and 0 for otherwise. This classical formulation of intervening opportunities as shown in Eq. (1) has the strong assumption described as $I\left( {d_{ij} > d_{ik} } \right)$, where the geographical extent is determined by distance $d_{ij}$. Several studies tackled the assumption using other criteria that substituted for distance^21,49,50^ and some parameters reflecting opportunities perceived by their geographical extent^49,51^.

The derivation of the radiation model with the separation term begins with considering the probability, $p_{ij}$, wherein an individual selects the destination $j$ from the origin $i$:

2 $$p_{ij} = \mathop \smallint \limits_{0}^{\infty } Pr_{{P_{i} }} \left( z \right)Pr_{{s_{ij} }} \left( { < z} \right)Pr_{{P_{j} }} \left( { > z} \right)dz$$

where $P_{i}$ is the number of opportunities at *i*, $Pr_{{P_{i} }} \left( z \right)$ is the probability that the maximum benefit obtained after $P_{i}$ samplings is exactly $z$, $Pr_{{s_{ij} }} \left( { < z} \right)$ is the probability that the maximum benefit obtained after $s_{ij}$ samplings is less than $z$, and $Pr_{{P_{j} }} \left( { > z} \right)$ is the probability that the maximum benefit after $P_{j}$ samplings is greater than $z$. As per Simini et al.^30^, we can obtain:

3 $$\begin{aligned} p_{ij} & = \mathop \smallint \limits_{0}^{\infty } Pr_{{P_{i} }} \left( z \right)Pr_{{s_{ij} }} \left( { < z} \right)Pr_{{P_{j} }} \left( { > z} \right)dz \\ & = \frac{{P_{i} P_{j} }}{{\left( {P_{i} + s_{ij} } \right)\left( {P_{i} + s_{ij} + P_{j} } \right)}} \\ \end{aligned}$$

Finally, $p_{ij}$ is independent of $z$ and $p\left( z \right)$. The trip distribution follows the multinomial distribution based on $p_{ij}$. Thus, we obtain the prediction formula which is equal to the average given by a binomial distribution with the normalisation factor for a finite system^36^ as:

4 $$\hat{T}_{ij} = O_{i} q_{ij} = O_{i} \frac{{p_{ij} }}{{\mathop \sum \nolimits_{k}^{n} p_{k} }} = O_{i} \frac{{\frac{{P_{i} P_{j} }}{{\left( {P_{i} + s_{ij} } \right)\left( {P_{i} + s_{ij} + P_{j} } \right)}}}}{{\mathop \sum \nolimits_{k}^{n} \frac{{P_{i} P_{k} }}{{\left( {P_{i} + s_{ik} } \right)\left( {P_{i} + s_{ik} + P_{k} } \right)}}}}$$

where $\hat{T}_{ij}$ is the prediction of the total number of trips from *i* to *j*, $q_{ij}$ is the normalised probability that an individual selects $j$ from $i$, and $O_{i}$ is the total number of trips departed from *i*.

The intervening opportunities $s_{ij}$ in the radiation model^30^ is equivalent to the classical definition of intervening opportunities^7^. Both the radiation and Schneider models commonly assume that when an individual searches for a proper destination to move to, they will select an area that is closest to the origin among locations with higher benefits compared to the origin. In the radiation model, the geographic extent of intervening opportunities is defined as the circle centred at the origin, with a radius the distance to the destination; and that a mover evaluates all locations in shorter-order from the origin of the extent. Finally, the mover selects the nearest location with a higher benefit compared to the origin.

**Figure 2:**
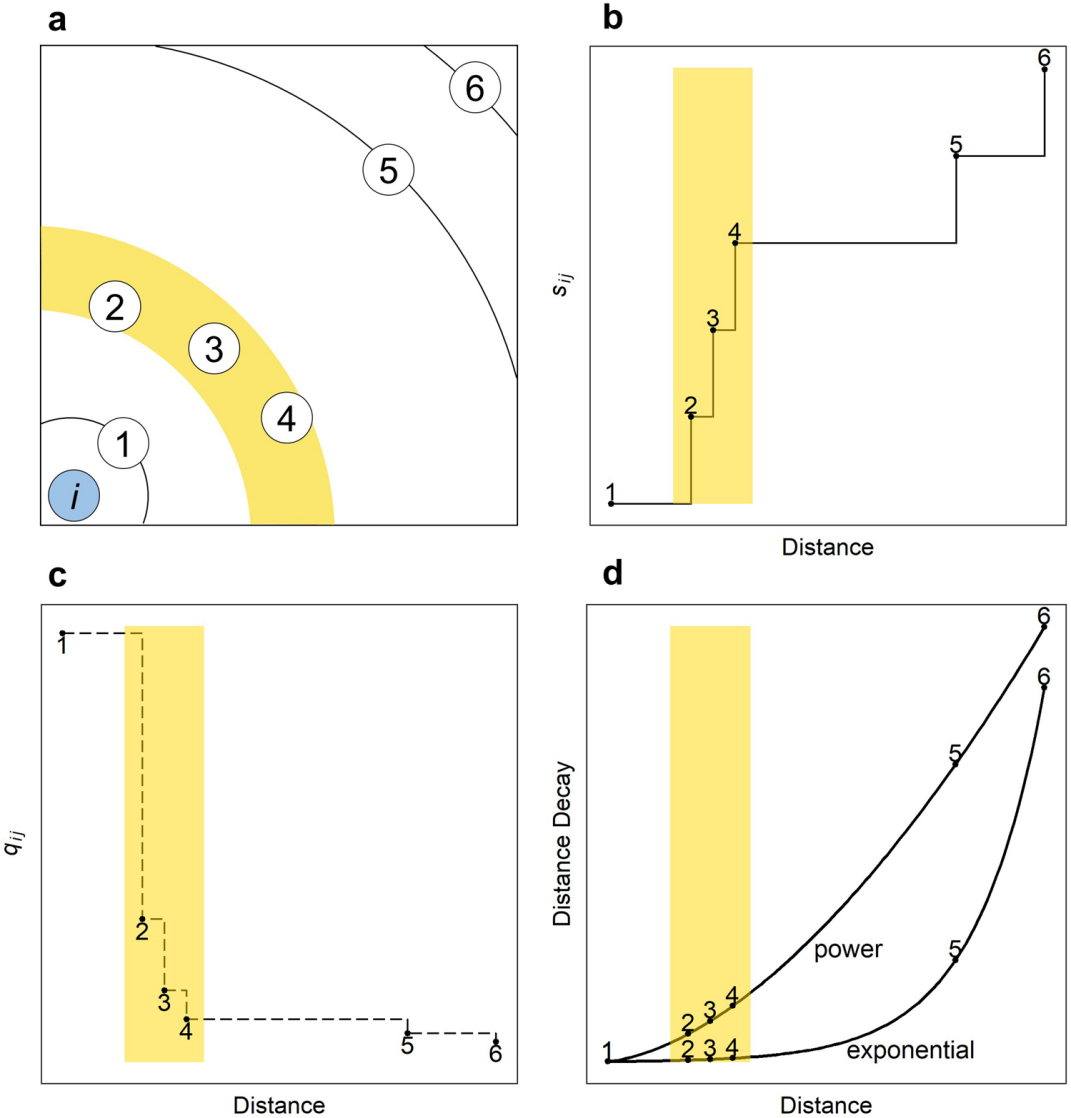
Location setting example and the changes in distance decay curves, $s_{ij}$, and $q_{ij}$. (**a**) The location setting example shows that areas 2, 3, and 4 locate almost the same distance from the origin. (**b**) The intervening opportunities for the setting. (**c**) The normalised probability $q_{ij}$ that an individual selects the destination* j* from origin *i* for the setting. (**d**) distance decay effect for the setting.

Notably, the final step in practice can cause significant errors for short trips when the location whose benefit is higher than the destination is located almost at the same distance, but slightly farther from the origin. For example, assume that there are six areas excluding the origin which have an equal population, and there are three areas that exist almost at the same distance from the origin, as shown in Fig. 2a. In such a case, intervening opportunities $s_{ij}$ and the normalised probability of moving to destination *j*, $q_{ij}$, seems like a step function as shown in Fig. 2b, c. The radiation model tends to distribute a main mass of flows to short-distance trips; in other words, in the area of shorter-order from the origin as shown in Fig. 2c. Furthermore, compared to the smooth distance decay curves of gravity models as shown in Fig. 2d, $q_{ij}$ in the form of an almost step shape is strongly affected by the change of distance, i.e., the moving up or down of rank.

The contrived point of assumption of the radiation model is the closure of the spatial search process if an individual finds the area having a higher opportunity than the origin. Here, we assume a situation in which the destination $j$ is the current primary candidate having higher benefit than the origin $i$, but there is a farther area $d$ having a higher benefit compared to the destination $j$, as illustrated in Fig. 1; this is discussed in the opportunity priority selection (OPS) model^43^. In this situation, a mover following the spatial search behaviour of the radiation model ignores $d$ and decides to move to *j*. However, in practice, it is expected that farther but nearby areas with higher benefits compared to $j$ may be selected by the individual with a higher probability, as same as the case within the geographical extent of the OPS model. Thus, upon adding those opportunities, the performance of the radiation model is expected to improve. Notably, ‘nearby’ is dependent on the mover’s cognitive assessment of distance; therefore, we introduce a kernel function of distance decay to represent the fuzziness of the geographic extent for intervening opportunities for movers. This study focused on the criterion of the extent as in previous studies^21,49,50^, but also on potential destinations located farther than them.

Therefore, we propose a new and simple modification of intervening opportunities by introducing a kernel-based fuzzy extent of destination search as follows:

5 $$\begin{aligned} F_{ij} & = s_{ij} + s_{ij}^{{\prime }} \\ & = \mathop \sum \limits_{{k \ne i,{ }j}}^{n} P_{k} I\left( {d_{ij} > d_{ik} } \right) + \mathop \sum \limits_{{k \ne i,{ }j}}^{n} w_{ijk} \left( {d_{ij} ,{ }d_{ik} } \right)P_{k} I\left( {d_{ij} \le d_{ik} } \right) \\ \end{aligned}$$

where $F_{ij}$ is a new intervening opportunity, $s_{ij}^{^{\prime}}$ is the opportunities of farther areas compared to destination $j$, and $w_{ijk} \left( {d_{ij} , d_{ik} } \right)$ is a weighting kernel function that varies from 1 to 0. The weighting kernel function indicates how likely a mover evaluates the locations which are further away from the destination $j$ in the search process of destinations. Thus, the areas weighted by the value of one are always considered as ‘intervening’; therefore, Eq. (5) can be rewritten as:

6 $$F_{{ij}} = \sum\limits_{{k \ne i,j}}^{n} {P_{k} } {\text{ }}w_{{ijk}}^{\prime } \left( {d_{{ij}} ,d_{{ik}} } \right)$$

where:

7 $$w_{{ijk}}^{\prime } \left( {d_{{ij}} ,d_{{ik}} } \right) = \left\{ {\begin{array}{*{20}l} {1,} \hfill & {d_{{ij}} \ge d_{{ik}} } \hfill \\ {w_{{ijk}} \left( {d_{{ij}} ,d_{{ik}} } \right),} \hfill & {otherwise} \hfill \\ \end{array} } \right..$$

Possible weighting functions for empirical analysis are discussed later.

### Incorporating ${\varvec{F}}_{{{\varvec{ij}}}}$ into the radiation model

We applied $F_{ij}$ to the spatial search behaviour of the radiation model. The probability that an individual selects the destination $j$ from origin $i$ is

8 $$p_{ij} = \mathop \smallint \limits_{0}^{\infty } Pr_{{P_{i} }} \left( z \right)Pr_{{F_{ij} }} \left( { < z} \right)Pr_{{P_{j} }} \left( { > z} \right)dz$$

where $Pr_{{F_{ij} }} \left( { < z} \right)$ is the probability that the maximum benefit obtained after $F_{ij}$ samplings is less than $z$.

Focusing on the spatial search behaviour, a mover assesses $P_{i}$, $F_{ij}$, and $P_{j}$ in order. Thus, in the model, it is assumed that when an individual searches for a destination to move to, an area will be selected that is closest to the origin, amongst destination candidates having higher opportunity benefits than those of the origin. However, $F_{ij}$ includes the opportunities of physically farther areas compared to the destination $j$. Considering the spatial search order, $p_{ij}$ can be rewritten as

9 $$p_{ij} = \mathop \smallint \limits_{0}^{\infty } Pr_{{P_{i} }} \left( z \right)Pr_{{s_{ij} }} \left( { < z} \right)Pr_{{s_{ij}^{^{\prime}} }} \left( { < z} \right)Pr_{{P_{j} }} \left( { > z} \right)dz$$

In this case, a mover assesses $P_{i}$, $s_{ij}$, $s_{ij}^{^{\prime}} ,$ and $P_{j}$, where $s_{ij}^{^{\prime}}$ is the newly introduced intervening opportunities by locations that are slightly farther compared to location *j*. The radiation model is based on the diffusion process of particles and their probability of being absorbed^30^. Equation (9) implies the following steps: (1) the particle is generated in $i$, (2) the particle has a probability of not being absorbed in a circle of centre $i$ with the radius of the distance between *i* and $j$, (3) the particle has a probability of not being absorbed in slightly further areas compared to *j*, and (4) the particle has a probability of being absorbed in $j$. Steps (3) and (4) violate the classical order of spatial search based on the physical distance. This violation can be interpreted that a mover may search for slightly further locations described by the kernel before deciding the destination $j$. This can occur through a cognitive misunderstanding or inadequate calculation of distance (e.g., using Euclidean distance when the network-based distance affects the movers’ evaluation of locations). It would be also possible to interpret that the violation may reflect our wavering nature— a kind of heuristics—of decision-making among comparable potential choices. Specifically, first choose $j$ by comparing other locations that are nearer to the origin, then attempt to confirm that the location $j$ is better compared to slightly further locations, and finally choose $j$ as the destination.

Following the derivation of the radiation model^30^, we obtain:

10 $$p_{ij} = \frac{{P_{i} P_{j} }}{{\left( {P_{i} + F_{ij} } \right)\left( {P_{i} + F_{ij} + P_{j} } \right)}}$$

and a prediction formula as:

11 $$\hat{T}_{ij} = O_{i} q_{ij} = O_{i} \frac{{p_{ij} }}{{\mathop \sum \nolimits_{k}^{n} p_{ik} }} = O_{i} \frac{{\frac{{P_{i} P_{j} }}{{\left( {P_{i} + F_{ij} } \right)\left( {P_{i} + F_{ij} + P_{j} } \right)}}}}{{\mathop \sum \nolimits_{k}^{n} \frac{{P_{i} P_{k} }}{{\left( {P_{i} + F_{ik} } \right)\left( {P_{i} + F_{ik} + P_{k} } \right)}}}}$$

### Weighting kernel functions

Regarding the functional form of the kernel, we employed two possibilities where $\mu$ and $\nu$ are parameters.

12 $$w_{ijk} \left( {d_{ij} ,d_{ik} } \right) = \left( {\frac{{d_{ij} }}{{d_{ik} }}} \right)^{\mu }$$

13 $$w_{ijk} \left( {d_{ij} ,d_{ik} } \right) = exp\left\{ { - \frac{ln2}{\nu }\left( {d_{ik} - d_{ij} } \right)} \right\}$$

**Figure 3:**
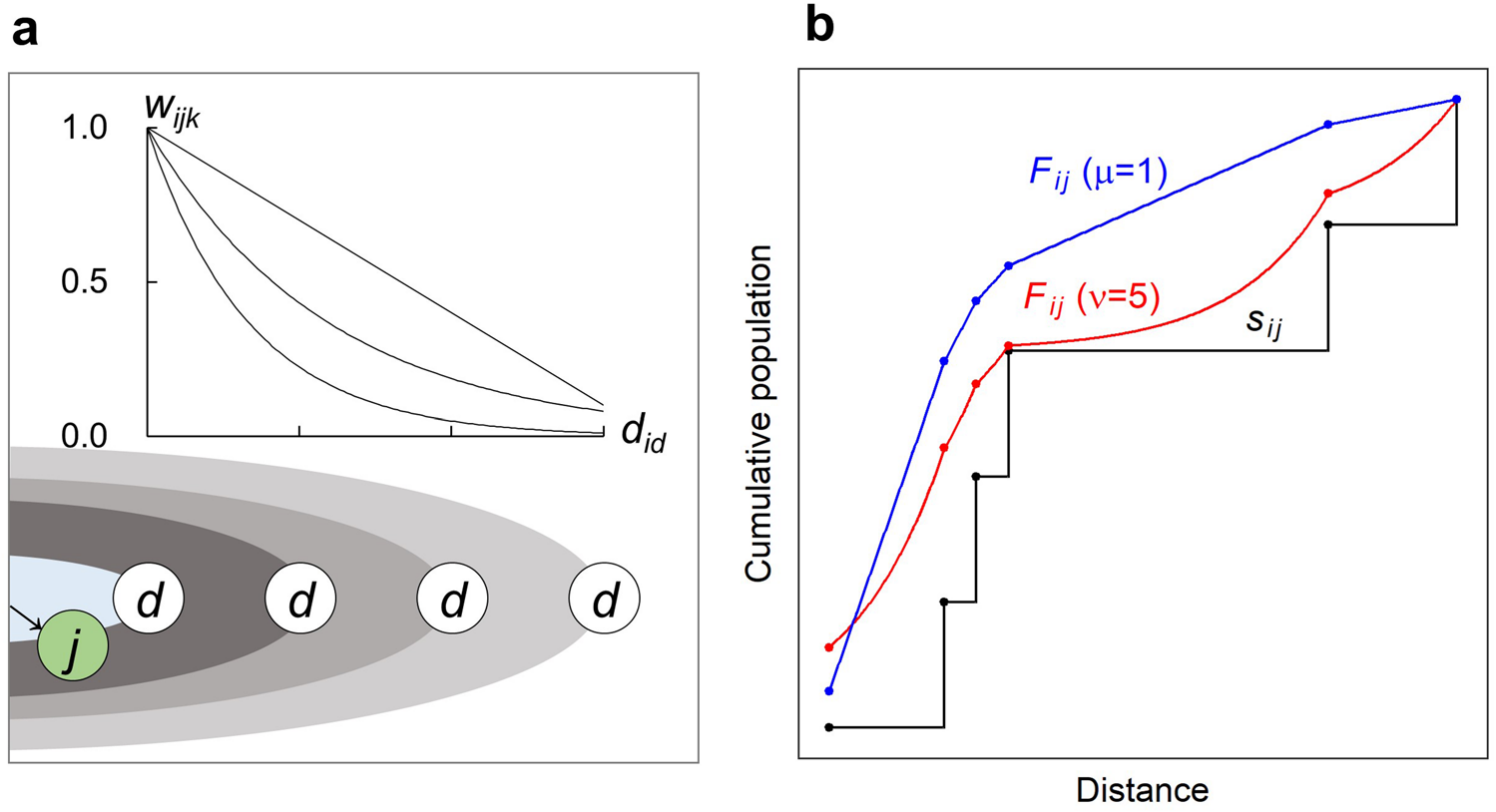
Conceptual illustrations of weighting kernel function and relationship between distance and cumulative population. (**a**) Illustration of weighting kernel function $w_{ijk}$. (**b**) Comparison among classical intervening opportunities $s_{ij}$, and two examples of $F_{ij}$ that are newly proposed those including the opportunities of farther areas compared to destination $j$ in the same setting of Fig. 2a.

Both functions take the value of 1 if $d_{ij} = d_{ik}$, and assume smaller values when $d_{ik}$ increases, as shown in Fig. 3a. The difference between Eqs. (12) and (13) reflects how movers perceive the farther opportunities at area $k$ differently, based on the distance from the destination $j$. $F_{ij}$ including the opportunities of outer areas if the extent is a smoother function of the distance from the origin by introducing the fuzziness of distance between $j$ and $k$, $w_{ijk} \left( {d_{ij} , d_{ik} } \right)$ as shown in Fig. 3b. If $\mu$ takes a large value or $\upsilon$ takes a low value, only the farther areas that are close to the border of the extent have strong effects of intervening opportunities by the kernel functions, and other areas scarcely influence movers.

**Figure 4:**
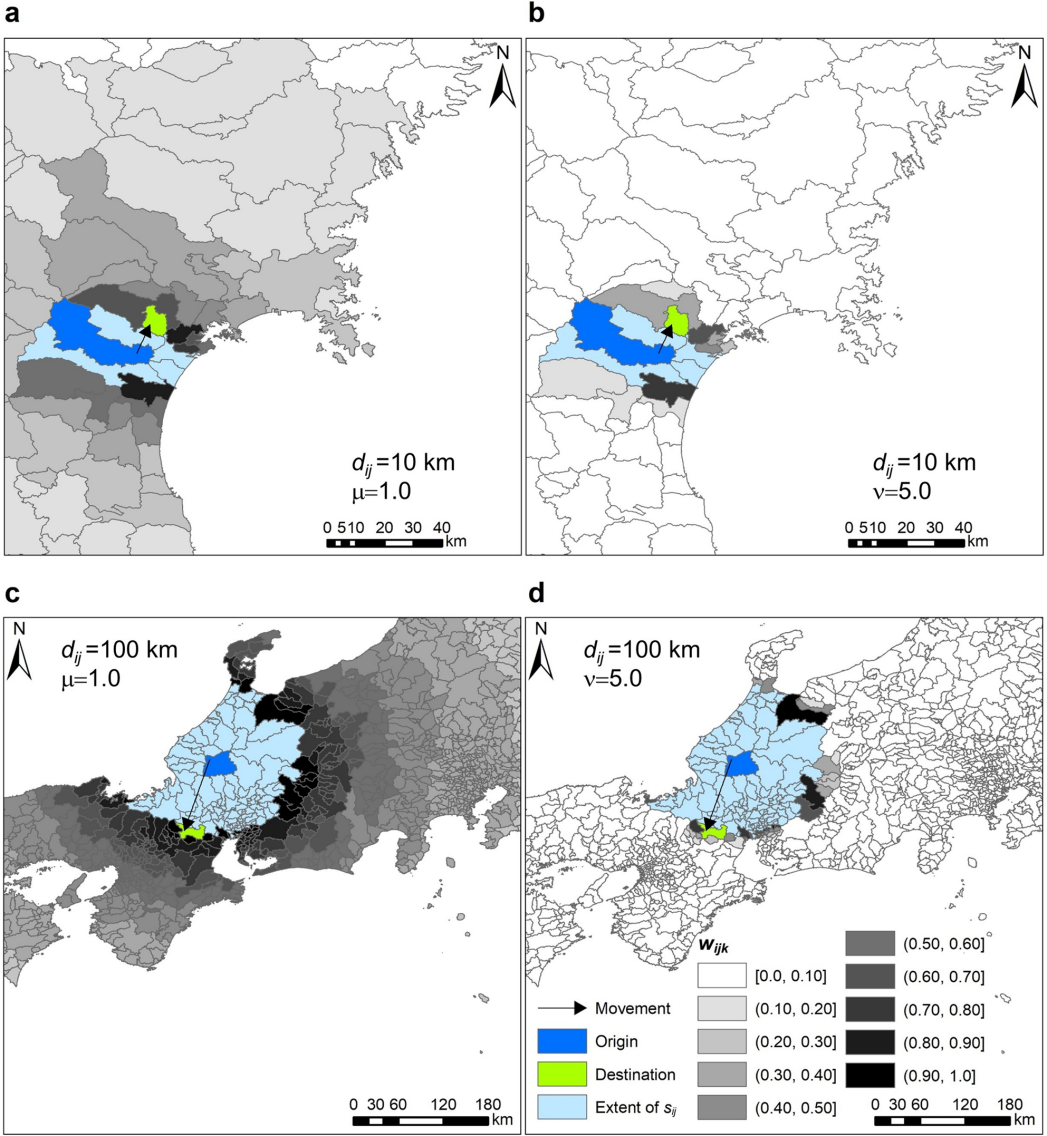
Empirical examples of weighting kernel functions based on Japanese municipalities and distance between population centres of those. (**a**, **b**) example in which the distance between the population centres of origin and destination is approximately 10 km, and (**a**) and (**b**) are corresponding to the kernel function in the case of $\mu = 1.0$ and $\nu = 5.0$, respectively. (**c**, **d)** same as (**a**, **b**), but the distance is approximately 100 km, and (**c**) and (**d**) are corresponding the case of $\mu = 1.0$ and $\nu = 5.0$ respectively. The map in this figure was created with ArcGIS version 10.7 (http://desktop.arcgis.com/).

Figure 4 shows empirical examples, describing how the weighting kernel works when $\mu = 1.0$ and $\nu = 5.0$. Figure 4a, b shows examples in which the distance between the population centres of origin and destination is approximately 10 km, and Fig. 4c, d shows examples in which the distance between the population centres of origin and destination is approximately 100 km. In addition, (a, c) of the figure is an example of the function where $\mu = 1.0$, and (b, d) is where $\nu = 5.0$.

Equation (12) adopts the ratio of the distances. It therefore shows that when an individual takes a trip to the destination ten kilometres away from the origin, the farther locations that are twenty kilometres away act as under half of the intervening opportunities to the individual. The same applies to a case when the destination is one hundred kilometres away from the origin and the farther locations are two hundred kilometres away. Therefore, we can interpret Eq. (12) as the logarithmic perception of travel cost, similar to the power function of the classical gravity model^52^. This assumes that the longer the distance is to the destination, the shorter the distances are to the farther areas, based on the mover’s sense of distance, as shown in Fig. 4a, c. Conversely, in Eq. (13), the difference in the distance and the exponential decay functions are used, where the denominator parameter $\nu$ indicates the distance required for opportunities to reduce to half the initial values. Equation (13) is not affected by the distance to the destination; therefore, it takes the same value under the condition when there is the same difference in distances, regardless of the value of the distances to destinations as shown in Fig. 4b, d.

### Parameters

Because the newly modified radiation model including $F_{ij}$ has one parameter $\mu$ or $\nu$, empirically observed mobility data are needed for estimating the parameter. Owing to the non-linear formulation, it is computationally difficult to apply a maximum likelihood estimation for the model. Thus, we attempted to include a series of specific numbers for the parameters in the model using four census mobility datasets before comparing it to other models. These three commuting datasets are those of the UK, the US, and Japan (hereafter called UK_C, US_C, and JP_C, respectively), and migration datasets of Japan (JP_M) are in Table 1. These datasets excluding US_C are the latest available examples, and the US_C is the same example as used by Simini et al.^30^ for demonstrating the radiation model’s performance. Following previous work^30,38^, the residential population and population centres or centroids of areas were used to measure opportunities and distances, respectively (all data are available online; see “Data availability”). In the analysis, we excluded the areas that have no inter-regional flows and residential populations. The values we evaluated were 1.0, 1.5, 2.0, 2.5, 3.0, 3.5, and 4.0 for $\mu$ and 1.0, 2.5, 5.0, 7.5, 10, 20, and 50 for $\nu$. The suitability of these values was evaluated via the Sørensen similarity index (SSI) and the percentage of deviance explained (Pdev; see goodness-of-fit indicators in the “Methods” section).

**Table 1:** Description of the datasets used to the newly modified radiation model and compare it other models.

Code	Country	Type	Number of units	Number of trips (migrants)	Average regional surface areas (km^2^)	Census year
US_C	US	Commuting	3140 counties	34,116,775	3129.5	2000
UK_C	UK	Commuting	404 local authorities	11,260,336	615.3	2011
JP_C	Japan	Commuting	1888 municipalities	28,106,916	196.0	2015
JP_M	Japan	Migration	1888 municipalities	13,300,422	196.1	2015

**Figure 5:**
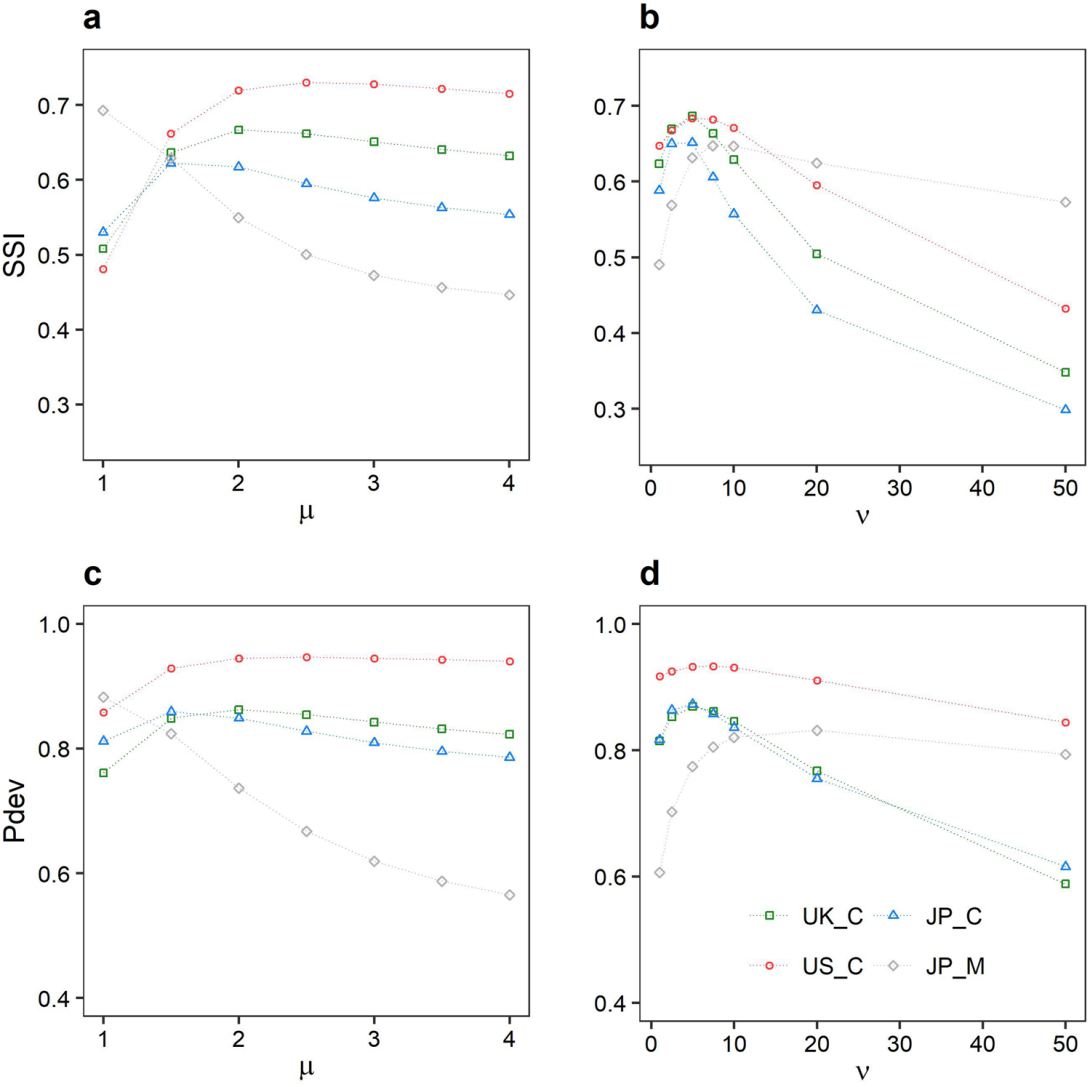
Relationship between proposed kernel-based radiation model’s parameters and goodness-of-fit indicators for UK_C, US_C, JP_C, and JP_M datasets. (**a**, **b**) Variation of SSI (Sørensen similarity index) for $\mu$ and $\nu$. (**c**, **d**) Variation of Pdev (Percent deviance explained) for $\mu$ and $\nu$. Note that the best parameters were $\nu$ = 5.0, $\mu$ = 2.5, $\nu$ = 5.0, and $\mu$ = 1.0, for UK_C, US_C, JP_C, and JP_M datasets, respectively, based on SSI, as with Pdev.

As a result, the preferable function and parameter values vary depending on the dataset. The result of the SSI evaluation is shown in Fig. 5a, b, and the best parameters were determined as $\nu$ = 5.0, $\mu$ = 2.5, $\nu$ = 5.0, and $\mu$ = 1.0 for UK_C, US_C, JP_C, and JP_M, respectively. The result obtained based on Pdev was similar to that based on SSI, as shown in Fig. 5c, d.

### Model predictions

**Figure 6:**
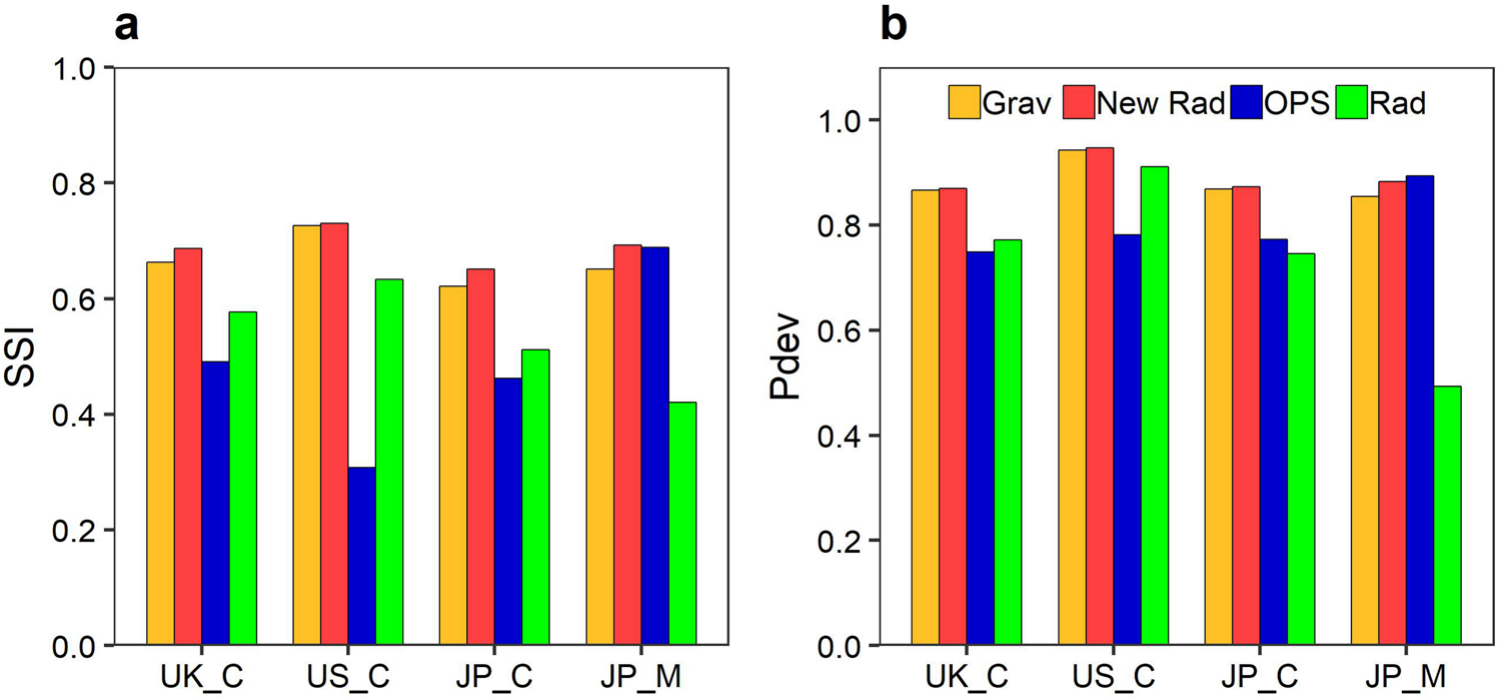
Comparison of accuracy of model fitting; (**a**) predictive accuracy of models based on SSI (Sørensen similarity index). (**b**) Same as (**a**) but based on Pdev (Percent deviance explained). Grav, New Rad, OPS, and Rad indicate the production-constrained gravity model, the kernel-based radiation model, the OPS model, and the original radiation model, respectively.

The newly proposed radiation model including $F_{ij}$ (Eq. (11)) (hereafter referred to as the kernel-based radiation model) was compared with the production-constrained gravity model, i.e., the opportunity priority selection (OPS) model, which is a parameter-free and universal model with a derivation like the radiation model^43^, and the original radiation model [Eq. (4)] using the four census datasets (see comparative models in the “Methods” section). Figure 6 displays the SSI and Pdev values obtained with each model. As expected, the kernel-based radiation model outperforms the original radiation model in all cases. The model surpassed or performed equally as the gravity model in all cases. Focusing on the universal models, the original radiation model performed poorly using the migration data, and the OPS model had poor predictive accuracy for the commuting data; therefore, the result shows that the universal models follow some specific flow corresponding to assumed spatial search behaviour of these models. Although the new model assumed the same behaviour as in the radiation model, the flexibility of the geographical extent of intervening opportunities based on the geographic kernel function may weaken the assumption and its effect.

**Figure 7:**
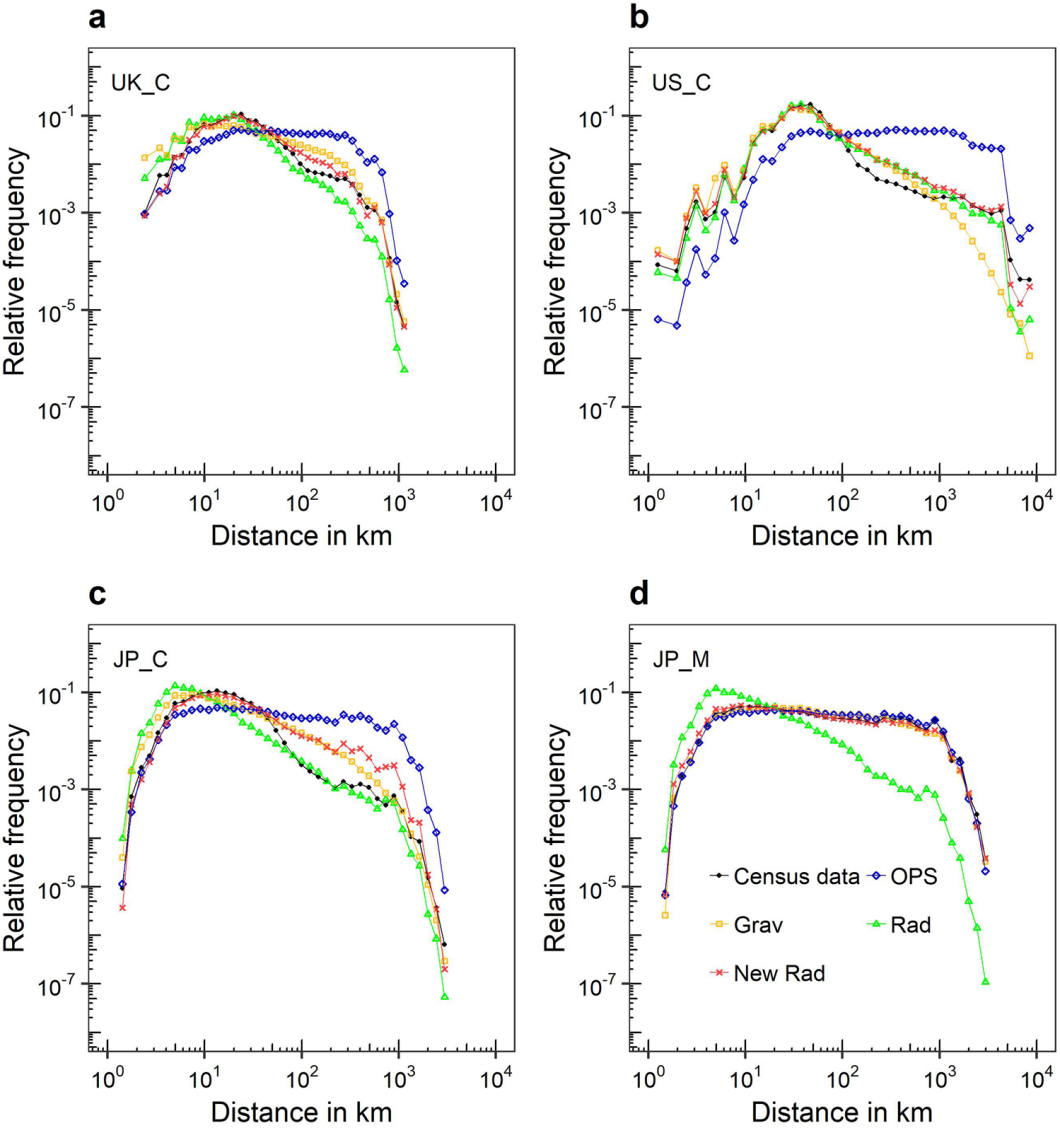
Relative frequency of trip size distribution by distances for census-based observations and model predictions by datasets: (**a**) UK_C. (**b**) US_C. (**c**) JP_C. (**d**) JP_M. Grav, New Rad, OPS, and Rad indicate the production-constrained gravity model, the kernel-based radiation model, the OPS model, and the original radiation model, respectively.

We investigated the trip distribution in the distance bands obtained by the models. This is a key quantity for measuring the accuracy of production-constrained mobility models such as the radiation model, as these models cannot ensure the agreement between the predicted travel to a location and the real travel to the same location. As shown in Fig. 7, the frequency distributions of travel distance predicted by the new model are in accordance with those of census data. Although the mobility type is different in Fig. 7c, d, the original radiation model and the OPS model showed similar distributions for both mobility types. The original radiation model at long distances sometimes predicted the flows accurately, as shown in Fig. 7b, c, and in other times it did not perform as well as the gravity model, as illustrated in Fig. 7a, d. A previous study showed that the radiation model and the gravity model with a power distance decay function can estimate commuting flows at large distances^32^, but the results may indicate that the predictive accuracy of those models depends on the datasets as well as the spatial scales.

The kernel-based radiation model at long distances performed like the gravity model, rather than the original radiation model, in three cases (Fig. 7a, c, d), and its predictive accuracy was lower than the original radiation model only in one case, as shown in Fig. 7c. Therefore, the newly proposed model does not necessarily resemble an accurate model at large distances because the main mass of the total size of flows is at short distances, especially in the commuting datasets as in the previous study^32^.

In addition, we explored the SSI distribution in the distance bands. As shown in Figure S1 (see Supplementary Information), although the original radiation model performed strongly at long distances in the cases of US_C and JP_C (see Fig. 7), the kernel based radiation model performed better than the original model at almost every distance band in all datasets. From the shape of the distribution of the model and SSI values, it is evident that the new definition of intervening opportunities improves on the faults of the original radiation model for predicting flow sizes at short and moderate distances^32,36,39,43,46^. Additionally, from these results and the comprehensive predictive accuracy, we observe that the radiation model can be improved by the kernel-based formulations of intervening opportunities.

## Discussion

In this study, we have proposed a new kernel-based formulation of intervening opportunities reflecting the decision-making process of a mover’s destination choice among multiple opportunities, including ambiguous areas that are farther than the final destination. The mobility patterns resulting from the kernel-based radiation model were in accordance with those of the four census datasets, with respect to the two model assessment indices and trip distance distributions. The results indicated that the new model captures more realistic mechanisms governing human movement compared to the classic models of intervening opportunities. Notably, the results suggested that the fuzziness of distances caused by the assessment of “nearby” is an essential factor of the spatial separation $S_{ij}$ for general spatial interaction modelling.

In previous studies, the radiation model has tended to overestimate short-distance flow and underestimate the long-distance flow at the city scale^34,43,46^. The kernel-based radiation model likely solves the overestimation for the flows over short distances at this scale, as shown in Fig. 7.

In predicting commuting flow volumes, the kernel functions were adopted to add the opportunities of the areas where the distance was almost the same as that of the destination, as shown in Fig. 4b, d. In predicting migration flow volumes, the function has a broad zone taking high values, as shown in Fig. 4a, c. In that case, the result indicated that the underlying mechanism of the decision-making process is not the same as the commuting flow. Migrants may tend to place high importance on the destination opportunity benefit rather than the distance to the destination as mentioned in a previous study^44^.

In the new model, before selecting the destination, a mover evaluates all areas in short-order from the origin in the geographical extent of intervening opportunities as well as the farther areas with the weighting kernel functions. Hypothetically, a two-stage process can be envisioned after selecting a destination; the mover may evaluate the farther areas to confirm if the selection is a satisfactory destination. This decision-making process has two possibilities for the selected destination: an individual accepts the first satisfactory opportunities at the destination $j$ as in the original radiation model, or they find a farther area which has higher benefits than $j$, and then they re-examine $j$ as an intervening opportunity. Both possibilities commonly assume that the finally selected destination has a higher benefit compared to those of $s_{ij}^{^{\prime}}$ in the evaluation process of intervening opportunities. From this viewpoint, the kernel-based radiation model may contribute to discussions of aggregate movement modelling and studies focusing on the spatial two-stage decision-making process^12,13,17,18^. Apart from intervening opportunities, the competing destinations model, which is an origin-constrained gravity type model with the term of accessibility of the destination, was proposed to consider spatial structures, locational patterns of origins and destinations in a two-stage decision-making process of destination choice^12,13^. A possibility to combine these terms based on different spatial decision-making processes, as in the previous studies using gravity models^11,17,18^, would be a topic to be explored as a next step.

There are some limitations to this study. First, to validate the effectiveness of a new formulation of intervening opportunities, a more detailed analysis is required for different types of flows and motivations, such as in consumer, tourism, and pleasure travel spatial behaviours. The framework of the radiation model and the newly proposed model are based on the job searching process; thus, additional explanations of the concept of opportunities, corresponding to a variety of interactions, are also necessary to consider. Focusing on the results of commuting datasets, the parameters of kernels might have relationships with the regional scale. For example, the parameter $\mu$ increased with the scale based on the highest values of goodness-of-fit indicators and the scales as shown in Fig. 5a, c and Table 1. Therefore, a comparative analysis using other datasets is needed to reveal the relationships between the parameters and the scales. Secondly, only two types of kernel functions were tested, and the empirically optimal values of those parameters were obtained from among prespecified interval values. Finally, the major difference of the kernel functions used in this study was whether the function includes the logarithmic perception similar to the power decay of the gravity model^53^. More comprehensive model constructions, including functions considering other perception of movers and suitable methods of parameter estimation, will be undertaken in future work.

In summary, the new kernel weighted formulation of intervening opportunities can express the different perceptions of farther opportunities, using two types of weighted kernel functions. Even though the preferable function and parameters were dependent on the geographical setting and flow types, the proposed model surpassed the original radiation model and outperformed or equally performed other models in all our evaluated cases in terms of statistical model performance. The difference in suitable functions and parameters in the empirical results may indicate the existence of other types of spatial search behaviour, which may provide new insights into spatial interaction modelling.

## Methods

### Comparative models

The production-constrained gravity model, the OPS model^43^, and the original radiation model^30,36^ were employed to compare the performance of the newly proposed model. The conventional production-constrained gravity model is defined as:

14 $$\hat{T}_{ij} = O_{i} q_{ij}$$

15 $$q_{ij} = \frac{{P_{j}^{\alpha } d_{ij}^{ - \beta } }}{{\mathop \sum \nolimits_{k}^{n} P_{k}^{\alpha } d_{ik}^{ - \beta } }}$$

where $\alpha , \beta$ are the parameters to be estimated. Because the distances between regions of the datasets are relatively large, the power function $d_{ij}^{ - \beta }$ is preferred in this study^10,53,54^. Here, we used the model following generalised linear modelling, in which the dependent variables are assumed to follow a Poisson distribution because this is suitable for count data modelling^55^. The model is shown as:

16 $$T_{ij} \sim Poisson\left( {\hat{T}_{ij} } \right)$$

17 $$\hat{T}_{ij} = exp\left( {\gamma_{i} + \alpha lnP_{j} - \beta lnd_{ij} } \right)$$

18 $$exp\left( {\gamma_{i} } \right) = O_{i} \frac{1}{{\mathop \sum \nolimits_{k}^{n} P_{k}^{\alpha } d_{ik}^{ - \beta } }}$$

where $\hat{T}_{ij}$ is the mean that is logarithmically linked to a linear combination of the logged independent variables. The gravity model is fitted by maximum likelihood estimation.

The last comparative model is the OPS model^43^, which is a parameter-free and universal model that assumes the individual at an origin *i* chooses a destination *j* when the opportunity benefit of *j* is higher than the maximum opportunity benefits of *i* and those of the intervening opportunities $s_{ij}$. Following the derivation of the radiation model, the probability is formulated as:

19 $$p_{ij} = \mathop \smallint \limits_{0}^{\infty } Pr_{{P_{i} + s_{ij} }} \left( z \right)Pr_{{P_{j} }} \left( { > z} \right)dz = \frac{{P_{j} }}{{P_{i} + s_{ij} + P_{j} }}$$

where $Pr_{{P_{i} + s_{ij} }} \left( z \right)$ is the probability that the maximum benefit obtained after $P_{i} + s_{ij}$ samplings is exactly $z$. The predictive formula is:

20 $$\hat{T}_{ij} = O_{i} q_{ij} = O_{i} \frac{{p_{ij} }}{{\mathop \sum \nolimits_{k}^{n} p_{ik} }} = O_{i} \frac{{\frac{{P_{j} }}{{P_{i} + s_{ij} + P_{j} }}}}{{\mathop \sum \nolimits_{k}^{n} \frac{{P_{k} }}{{P_{i} + s_{ik} + P_{k} }}}}$$

Although several parameter-free and universal models for human mobility are proposed^30,38,43,46^, we used the OPS model for comparison to the newly proposed model, because the model has the derivation and high predictability regardless of spatial scale such as inter-city and intra-city scales^43,44^.

### Goodness-of-fit indicators

To assess the robustness of the results of each model, two indicators—the Sørensen similarity index (SSI) and the percentage of deviance explained (Pdev)—were used. The former, based on Sørensen’s index^56^, has been recently used as the criterion for model comparisons^33,36,42^ and it is defined as:

21 $$SSI = \frac{{2\mathop \sum \nolimits_{i} \mathop \sum \nolimits_{j} min\left( {\hat{T}_{ij} ,{ }T_{ij} } \right)}}{{\mathop \sum \nolimits_{i} \mathop \sum \nolimits_{j} \hat{T}_{ij} + \mathop \sum \nolimits_{i} \mathop \sum \nolimits_{j} T_{ij} }}$$

where $min\left( {\hat{T}_{ij} , T_{ij} } \right)$ is the function that returns the lower value of $\hat{T}_{ij}$ or $T_{ij}$. SSI varies from 0 to 1. Its value is 0 when there is no match between empirical data and modelled data, and 1 when there is a complete match. We assumed that the total size of the flow generated by the model follows a Poisson distribution, like classical gravity models^55^, shown as $T_{ij} \sim Poisson\left( {\hat{T}_{ij} } \right)$. Therefore, we use the following as a likelihood-based goodness-of-fit indicator, percentage of deviance explained, or pseudo R squared, Pdev:

22 $${\text{Pdev}} = 1 - \frac{{D_{1} }}{{D_{0} }}$$

where $D_{1}$ is the deviance of the model and $D_{0}$ is one of the null models which follows $\hat{T}_{ij} \sim Poisson\left( {O_{i} \frac{1}{n}} \right)$. The indicator ranges from 0 to 1, and a larger value indicates a better fit to the observed data.

### Data availability

In this study, all datasets used (mobility flows, population and population centres or centroids) can be obtained from census websites in each country. The 2000 US dataset can be found in the link [https://www.census.gov/population/www/cen2000/commuting/index.html] and [https://www.census.gov/geographies/reference-files/2000/geo/2000-centres-population.html]. The 2011 UK dataset is available at [https://data.london.gov.uk/dataset/place-residence-place-work-local-authority], [https://www.nomisweb.co.uk/census/2011/ks101uk] and [https://borders.ukdataservice.ac.uk/easy_download_data.html?data=infuse_dist_lyr_2011]. The 2015 Japanese commuting dataset can be downloaded at [http://www.stat.go.jp/data/kokusei/2015/kekka.html], and [http://www.stat.go.jp/data/kokusei/topics/topi102.html]. The 2015 Japanese migration dataset is online, and available at [http://www.stat.go.jp/data/kokusei/2015/kekka.html] (which is the same source of the commuting dataset), and [http://www.stat.go.jp/data/kokusei/topics/topi61.html].

## Supplementary Information

Supplementary Figure.
