# Supplementary material for: Kernel-based formulation of intervening opportunities for spatial interaction modelling
Source: Sci Rep. 2021 Jan 13;11:950. doi: 10.1038/s41598-020-80246-9 (PMC7807028; doi:10.1038/s41598-020-80246-9)
Supplement: Supplementary file 1 — Supplementary Figure. [file 41598_2020_80246_MOESM1_ESM.pdf]

Supplementary Information

Kernel-Based Formulation of Intervening Opportunities for Spatial Interaction Modelling

Masaki Kotsubo[1*] and Tomoki Nakaya[1]
[1]Graduate School of Environmental Studies, Tohoku University, Sendai, Japan

*Corresponding author, Email: masaki.kotsubo.s3@tohoku.ac.jp

Contents

S1 Goodness-of-fits of fitted models by distance bands 2

S1 Goodness-of-fits of fitted models by distance bands

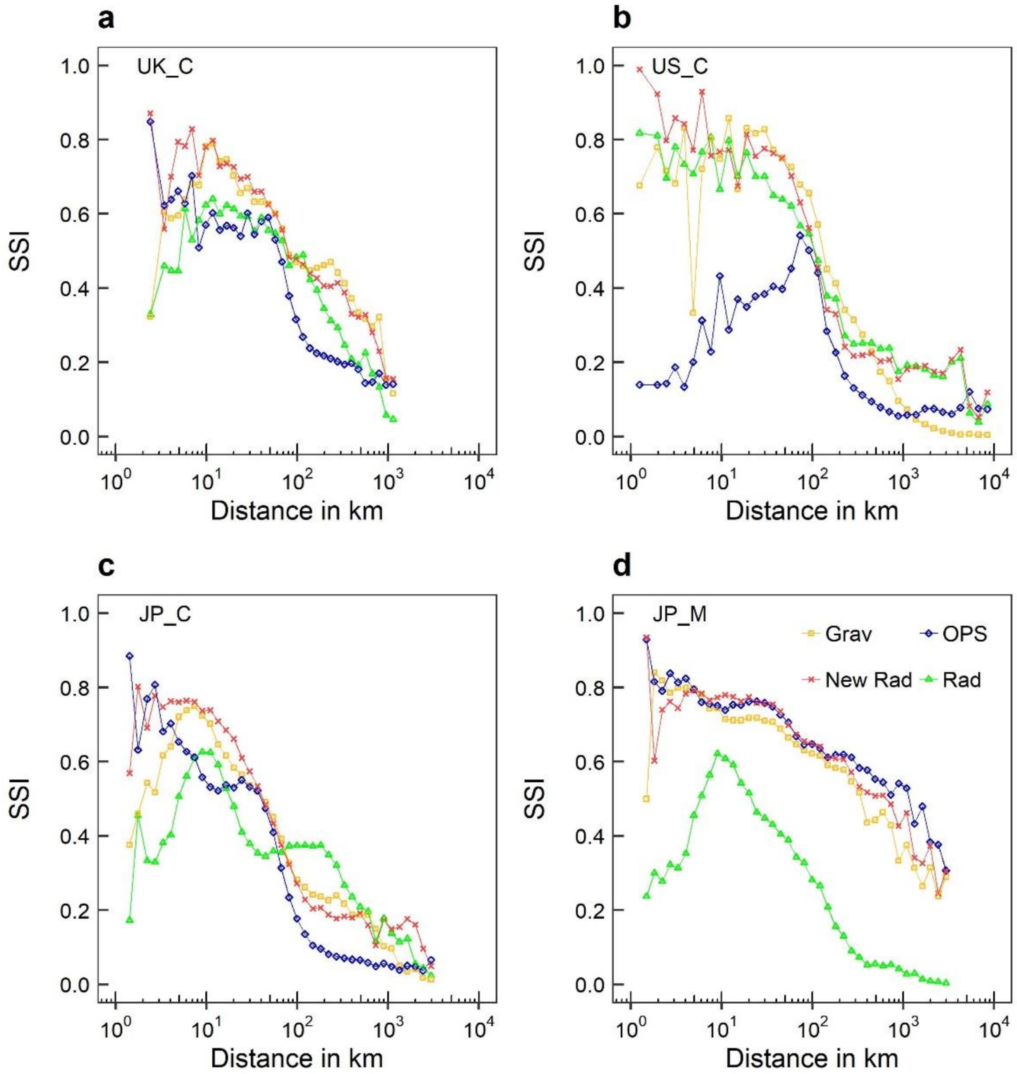

Figure S1. SSI of fitted models by distance bands for the four evaluated datasets: (**a**) UK_C, (**b**) US_C, (**c**) JP_C, and (**d**) JP_M. Grav, New Rad, OPS, and Rad indicate the production-constrained gravity model, the kernel-based radiation model, the OPS model, and the original radiation model, respectively.
